# Deciphering the Role of Colicins during Colonization of the Mammalian Gut by Commensal *E. coli*

**DOI:** 10.3390/microorganisms8050664

**Published:** 2020-05-02

**Authors:** Amanda N. Samuels, Manuela Roggiani, Kathryn A. Smith, Jun Zhu, Mark Goulian, Rahul M. Kohli

**Affiliations:** 1 Department of Veterinary Medicine, University of Pennsylvania, Philadelphia, PA 19104, USA; samuelsa@vet.upenn.edu; 2 Graduate Group on Cell and Molecular Biology, Perelman School of Medicine, University of Pennsylvania, Philadelphia, PA 19104, USA; junzhu@pennmedicine.upenn.edu; 3 Department of Biology, School of Arts and Science, University of Pennsylvania, Philadelphia, PA 19104, USA; roggiani@sas.upenn.edu (M.R.); kiikyo@gmail.com (K.A.S.); goulian@sas.upenn.edu (M.G.); 4 Department of Biology, Solenis LLC., Wilmington, DE 19803, USA; 5 Department of Medicine, Perelman School of Medicine, University of Pennsylvania, Philadelphia, PA 19104, USA

**Keywords:** colonization, colicin, commensal *E. coli*, DNA damage response

## Abstract

Colicins are specific and potent toxins produced by *Enterobacteriaceae* that result in the rapid elimination of sensitive cells. Colicin production is commonly found throughout microbial populations, suggesting its potential importance for bacterial survival in complex microbial environments. Nonetheless, as colicin biology has been predominately studied using synthetic models, it remains unclear how colicin production contributes to survival and fitness of a colicin-producing commensal strain in a natural environment. To address this gap, we took advantage of MP1, an *E. coli* strain that harbors a colicinogenic plasmid and is a natural colonizer of the murine gut. Using this model, we validated that MP1 is competent for colicin production and then directly interrogated the importance of colicin production and immunity for MP1 survival in the murine gut. We showed that colicin production is dispensable for sustained colonization in the unperturbed gut. A strain lacking colicin production or immunity shows minimal fitness defects and can resist displacement by colicin producers. This report extends our understanding of the role that colicin production may play for *E. coli* during gut colonization and suggests that colicin production is not essential for a commensal to persist in its physiologic niche in the absence of exogenous challenges.

## 1. Introduction

The intestinal microbiota is composed of an incredibly diverse array of commensal microbes that exist in a delicate yet relatively stable equilibrium. The stability and resilience of the healthy microbiome is critical for establishing a protective barrier from enteric pathogens [1], liberating essential nutrients [2], and modulating the host immune system [3]. Understanding the effectors that help to establish and maintain commensal colonization in the healthy gut is therefore critical, given the integral role of commensals in regulating gut homeostasis and host health.

In the complex intestinal environment, survival involves competition for space and nutrients [4,5]. Bacteria have therefore evolved mechanisms to both respond to environmental challenges and to counteract competitors fighting for the same resources [6,7,8]. One such mechanism that is environmentally responsive and serves to inhibit the growth and survival of competitors involves the production of diffusible antimicrobial molecules known as bacteriocins [9,10]. Bacteriocins are toxins that have a specific host range and are generally lethal to closely related strains competing for similar resources [11]. A subtype of bacteriocins, known as colicins, are produced by *Enterobacteriaceae*. Colicins and their related genes are found within genomic clusters on colicinogenic plasmids. These clusters typically contain the colicin production gene which codes for the toxin, an immunity gene which codes for a protein that confers self-resistance by binding to and inactivating the toxin protein, and a lysis gene which codes for protein that aids in colicin release [12]. In vitro experiments have demonstrated that colicin expression from these plasmids is tightly regulated by the bacterial DNA damage stress response pathway, also known as the SOS response, or by nutrient limitation [13,14,15]. 

Theoretical and laboratory-based studies have suggested that during periods of environmental stress, such as genotoxic stress or nutrient depletion, colicins aid in promoting survival of the colicin producing strain, potentially by modulating the broader microbial community [16,17,18]. Laboratory studies have defined synthetic triggers that precipitate colicin production, yet natural environments that trigger colicin production and subsequently require colicin production for bacterial survival have yet to be elucidated. One natural environment in which colicins may be instrumental for bacterial survival is the gastrointestinal tract. Colicin-producing strains are highly prevalent within the mammalian gut [12,19], with estimates that at least half of the *E. coli* strains in human fecal samples produce one or more colicins, and more than half of *E. coli* isolates from feral house mice contain colicinogenic plasmids [20,21]. 

The mammalian gut is characterized by a high density and diversity of microbes, making it a plausible environment in which colicins could be critical for successful commensal colonization. Beyond the high density of microbes, the potential for genotoxic stressors or periods of nutrient limitation suggest a potential role for colicin production. A recent report revealed that a functional SOS response is critical for a colicin-producing commensal *E. coli* isolate to successfully colonize the healthy mammalian gut [22]. While the SOS response provides many diverse functions for bacteria, noncanonical functions such as colicin regulation could be the critical effectors of the SOS response that are required for sustained colonization of the mammalian gut. Despite implicit evidence that colicins may be important for survival in the mammalian gut, little work has been done that directly tests whether colicins are critical for native commensals to sustain colonization in their natural environmental niche.

Attempts to elucidate the role of colicins within the intestinal microbiome have largely relied upon one of two mouse models in which the indigenous microflora and the homeostatic gut environment have been disrupted. In one model, streptomycin-resistant strains are used and streptomycin is administered throughout the course of the experiment. In this model, administering streptomycin is necessary to overcome colonization resistance, but continual streptomycin treatment has been demonstrated to reduce microbial diversity and alter the mouse immune status [23,24,25]. In a second model, colitis is induced to trigger expansion of strains; however, the factors involved in colonization likely differ in colitis relative to the unperturbed gut. These model systems have largely been employed because the commonly utilized *E. coli* laboratory strains are not natural colonizers of the murine gut and, consequently, in the absence of streptomycin or dysbiosis, most strains poorly and transiently colonize the healthy murine gut [23,26,27]. Despite the inherent limitations of these models, collectively these studies have suggested the importance of colicin production for stable colonization [28], for displacement of a colicin-sensitive strain [29], and for survival during intestinal competition [26,30,31] in the setting of dysbiosis. Whether colicin production is vital to commensal colonization in the context of a healthy gut environment and in the absence of antibiotic selection remains unknown. 

Given the limitations of prior models, we aimed to understand whether colicin production is a tool used by commensal bacteria to initiate and sustain colonization in a healthy gut with an intact microbiome. To address this gap, we studied the mouse intestinal commensal *E. coli* isolate, MP1, which can sustain colonization in the absence of exogenous antibiotic treatment [32]. MP1 has a naturally occurring colicinogenic plasmid, making it an ideal model to explore colicin dynamics during commensal colonization of the healthy gut environment. We demonstrate that colicin production is not critical for sustained colonization and that, in direct intestinal niche competition, colicin production does not confer a fitness advantage. Additionally, we found that an established commensal does not require colicin to limit invading strains. Our results suggest that although colicins may have a role in perturbed environments, host-adapted strains may not rely on colicin production for establishment and maintenance of colonization in a homeostatic environment. Our work lays the groundwork for further exploration into the environments that necessitate colicin production for bacterial survival and provides insight into molecular mechanisms that commensals use to sustain colonization in a healthy gut microbiota. 

## 2. Materials and Methods 

### 2.1. Isogenic Strain Construction

The mCherry-marked MP1 (also known as MP7) and the GFP-marked MP1 (also known as MP13) were previously described and used for competition experiments [22,32]. MP13 was used for strain construction as well as competition experiments [32]. 

The colicin-deficient strain, ∆*colY*, was constructed by deleting the *colY* gene via recombineering as described in reference [33], except that electrocompetent cells were prepared by washing with an ice-cold solution containing 20% glycerol and 1 mM unbuffered 3-(*N*-morpholino) propanesulfonic acid (MOPS) (Sigma, M3183, St. Louis, MO, USA) [32]. Appropriate primers with 50 bp of flanking homology to the *colY* locus were used to PCR amplify the kanamycin cassette and flanking FRT sites from pKD13 [33]. The integration of the cassette removed all of *colY* except the start codon and the last seven codons, as described [33]. The *colY* gene is on a plasmid and care was taken to remove the wild-type (WT) plasmid by re-streaking the strain on increasing concentrations of kanamycin. The colonies were screened by PCR for loss of the WT plasmid. The colicin-deficient and sensitive strain, ∆*colY* ∆*cyi*, was constructed in a two-step process starting with the removal of the kanamycin-resistant cassette from the Δ*colY* strain using FLP recombinase by transformation with pCP20 [33]. The *cyi* gene was deleted via recombineering by replacing it with a kanamycin resistance cassette as described above for the construction of Δ*colY*, with the difference being that the PCR product from pKD13 did not include the FRT sites. This was done in order to avoid integration of the PCR product in the existing FRT site left in *colY* after the removal of the Kan cassette. The integration of the kanamycin cassette removed most of *cyi* except the stop codon and the last 11 codons. The deletion was verified by PCR. The SOS-deficient MP1 strain was described previously and used for the GFP reporter assay [22]. 

### 2.2. GFP-Reporter Assays

Induction of the colicin promoter following DNA damage was monitored by constructing a reporter plasmid containing GFP under the control of the *colY* promoter. This GFP reporter construct was constructed in a manner similar to the library of GFP reporter plasmids developed by Zaslaver et al. [34]. To construct this plasmid, we amplified a 450 bp region of the MP1 genome that included 300 bp upstream of the SOS-binding boxes of the MP1 colicin gene and 150 bp into the colicin-coding region. This DNA fragment was cloned into the vector pUA66 and the construct was sequenced. Additional reporter plasmids contained GFP under the control of the *recA* promoter or *dinB* promoter as described previously [35]. Briefly, each bacterial strain was transformed with the GFP-reporter plasmids and cultured in minimal media containing 1 X M9 salts (Sigma M6030, St. Louis, MO, USA), 0.4% glucose, 2 mM MgSO_4_, 0.1 mM CaCl_2_, 0.05% Casamino acids, and 30 mg/mL of kanamycin for plasmid maintenance. Overnight cultures were diluted 1:1000 into fresh media and incubated at 37 °C with shaking until reaching an optical density at 595 nm of ~0.3. Then, 100 mL aliquots were dispensed into 96-well, round-bottom, transparent plates. DNA damage was induced by UV light (50 J/m^2^). Plates were incubated at 37 °C under agitation and GFP fluorescence (Ex/Em: 485 nm/535 nm) and culture density (OD_595_) was monitored continuously in 5 min intervals for 3 h. To prevent evaporation, 50 mL of mineral oil (Sigma, M5904, St. Louis, MO, USA) was added to each well. The level of promoter induction was determined by taking the ratio of the fluorescence intensity and the optical density (FI/OD). 

### 2.3. Colicin Production Assays

Colicin lysates were prepared by using a standard colicin production protocol [36]. Briefly, strains were grown overnight in LB broth at 37 °C shaking. Overnight cultures were diluted 1:100 into fresh LB media and grown to an OD_595_ of approximately 0.2–0.3. At this point, 1.0 mL of culture was transferred to a microcentrifuge tube, spun at 9000 rpm for 5 min, and resuspended in 0.5 mL of 10 mM MgSO_4_. Cells were transferred to 24-well plates and exposed to UV light. Following exposure to UV light, 0.25 mL of cells were transferred to 2.0 mL of LB, covered in aluminum foil, and incubated at 37 °C shaking for 3 h. Following incubation, 100 µL of chloroform was added and tubes were transferred to microcentrifuge tubes, vortexed, and spun in a microcentrifuge at 9000 rpm for 10 min. Following centrifugation, the supernatant was transferred to a clean glass tube and stored at 4 °C. For lysate preparation in the absence of UV light, the steps remained the same except without exposure to UV light.

### 2.4. Colicin Screening Assays

All strains used in this assay are described in Table S1. Screening assays were performed by growing each strain in LB broth at 37 °C with shaking. Indicator lawns for each strain were prepared by adding 50 µL of the cells (~1 × 10^9^ cells/mL) to 3.5 mL of top-agar, gently mixing, and then pouring onto LB agar plates. After the lawns solidified, 8 µL of lysate was spotted on the lawn in serial dilutions. All indicator strains and lysates were assayed in duplicate. 

### 2.5. Animal Experiments

All animal studies were carried out in accordance with guidelines of the Institutional Animal Care and Use Committee of the University of Pennsylvania. Animal protocols followed the guidelines established within the “Guide for the Care and Use of Laboratory Animals” published by the National Research Council of the National Academies (IUCAC-804529, 06-2012). Experiments were performed with 6- to 8-week-old C57BL/6 male mice purchased from Charles River Laboratories. All mice were fed standard rodent chow ad lib (LabDiet 5001, Ft. Worth, TX, USA) and had access to fresh water. Each cage contained 4 to 5 mice. The colonization protocol has been described previously [22], but briefly mice were given 5 g/L of streptomycin (Sigma, S6501, St. Louis, MO, USA) and glucose in their drinking water for 72 h. After 72 hrs, mice were given fresh water for 24 h prior to oral inoculation with *E. coli* strains and were maintained on fresh water throughout the remainder of the experiments. The inoculum preparation was also described previously [22], but briefly, cells were prepared by streaking out on LB agar plates and incubating at 37 °C overnight. The next day, a single colony was picked and grown overnight in LB broth shaking at 37 °C. The following day, optical density at 595 nm was measured and the concentration of cells was calculated. Cells were spun down at 3800× *g* at 4 °C and resuspended in cold Phosphate Buffered Saline (PBS). Cells were washed twice with PBS and resuspended to a final cell density of ~10^10^ to 10^11^ cells/mL. For the competition experiment, cell suspensions were mixed at a 1:1 ratio and mice were orally inoculated with ~100 µL of the suspension. For the solo-colonization experiment, ~100 µL of each strain was given to each mouse. For the pre-colonization experiment, mice were solo-colonized as previously described. Then, 9 days post colonization, mice were orally inoculated with 100 µL of either ~10^7^ cell/mL of the wild-type strain or ~10^7^ cells/mL of the ∆*colY* ∆*cyi* strain. Each inoculum was prepared as previously described. 

To determine the colony counts, at each time point 3 to 4 fresh fecal pellets were obtained from each mouse [32]. Fecal samples were weighed and resuspended to reach a final concentration of 0.5 g of feces per 1.0 mL of PBS. The samples were vortexed and incubated at 4 °C for roughly 1 h. The samples were serially diluted in PBS and plated on LB agar plates containing 15 µg/mL tetracycline. Fluorescence images of the plates were obtained as described previously [37] and the colony forming units (CFU) were determined. The competitive index (CI) was determined as [(mCherry fluorescent CFU)/(GFP fluorescent CFU)]/[(input mCherry CFU/input GFP CFU)], where the initial inoculum represents input CFU.

## 3. Results

### 3.1. Regulation and Functionality of the MP1 Colicin

**Figure 1:**
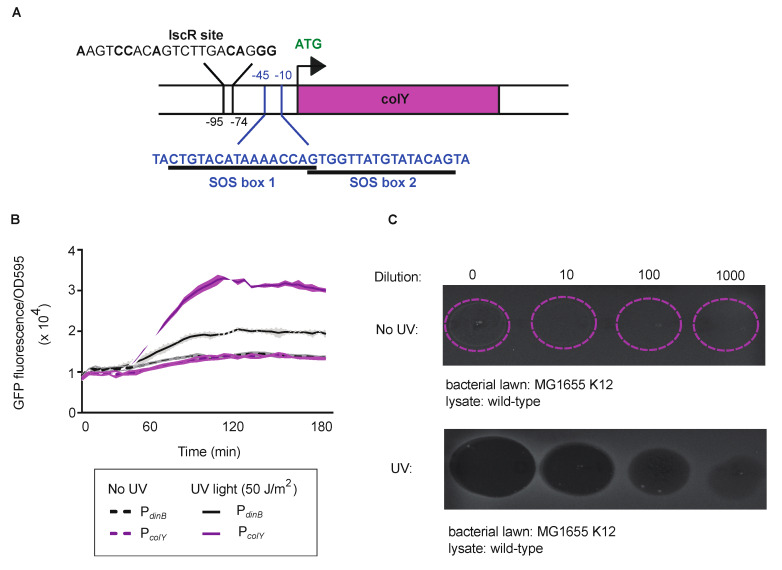
The MP1 colicin is SOS (bacterial DNA damage stress response pathway) regulated and active. (**A**) Sequence analysis revealed two overlapping SOS boxes and an IscR binding site upstream of the start codon. (**B**) GFP expression from the colY promoter depends upon DNA damage. The kinetics of GFP expression mirror another SOS-controlled gene, *dinB*. Time-dependent induction of GFP is represented as fluorescence intensity normalized to optical density at 595 nm at the start of the experiment. The error bands show the standard deviations of results from four independent biological replicates for each condition. (**C**) Shown is the inhibition halo of *E. coli* K12 (MG1655) by cell lysates of wild-type MP1 generated either with or without UV exposure. Dilutions of cell lysates were made and indicated. Dashed circles indicate location of spotted lysate.

MP1 offers the opportunity to explore whether colicins are important for a commensal bacterial strain living within a healthy gastrointestinal tract [32]. MP1 harbors a 8.5-kb colicinogenic plasmid that carries a colicin gene (*colY*) with substantial homology to previously characterized pore-forming colicins, colicin Y and colicin U, as well as genes with homology to lysis (*cyl*) and immunity (*cyi*) proteins (Figure S1) [38,39]. In *E. coli*, the expression of many colicins is triggered by DNA damage and regulated by the SOS response [12,13,15]. In the SOS response, transcription is repressed by LexA which binds to the promoters of SOS-controlled genes. In the presence of DNA damage, LexA undergoes self-cleavage, repression is relieved, and transcription can occur. Genetic analysis of the promoter region of the MP1 colicin gene suggested two potentially overlapping canonical SOS boxes where LexA could bind (Figure 1A). Additionally, the MP1 colicin-promoter region contains an iron–sulfur cluster regulator (IscR) binding site upstream of the SOS boxes, suggesting another possible layer of colicin regulation [40]. IscR functions as a transcriptional repressor and dual repression of colicins with both LexA and IscR has been previously described in other colicins [41]. 

To first explore the responsiveness of the colicin promoter (P*_colY_*) to DNA damage, we engineered a reporter plasmid which places the green fluorescent protein gene (*gfp*) under the control of the MP1 P*_colY_* and introduced the plasmid into MP1. As a control, we placed *gfp* under the control of another well-studied SOS-inducible promoter from *dinB* (P*_dinB_*) [34]. In the absence of UV light as a DNA-damaging agent, no expression of GFP was observed for either construct. Upon exposure to UV light (50 J/m^2^) we observed inducible expression of GFP from both reporter constructs (Figure 1B). SOS-controlled genes also display time-dependent kinetics, with some genes being induced early in the SOS response, such as *recA*, and others being induced late in the response, such as *dinB* [35,42]. The GFP expression pattern of the *colY* promoter mirrored the expression pattern of the *dinB* promoter, suggesting there is a delay in *colY* induction from the initial activation of the SOS response, similar to some previous analysis of SOS-controlled colicins [43]. To verify the timing kinetics, we repeated the experiment in the presence of the SOS-inducible promoter, P*_recA_* (Figure S2A). GFP induction from the P*_recA_* construct was both higher in amplitude and faster than P*_colY_*, reinforcing the conclusion that colicin production occurs later in the SOS response. To verify that the expression of *gfp* from the colicin promoter was due to LexA cleavage and SOS induction, we utilized a previously constructed MP1 strain that cannot activate the SOS response due to inactivation of LexA self-cleavage [22,44,45]. As predicted, we no longer observed GFP expression in the presence of UV light (Figure S2B). Additionally, we noted a slightly higher baseline level of GFP expression in the wild-type strain relative to SOS-off strain, which suggests that spontaneous induction of the SOS response in a small minority of cells may contribute to basal colicin expression [46]. Collectively, these data demonstrate that MP1′s colicin is regulated by the SOS response, signifying that the MP1 colicin might be important for MP1 survival in the presence of genotoxic stress. 

To validate whether the MP1 model might prove useful for evaluating the role of colicin in vivo, we next aimed to explore the functionality of the MP1 colicin and to characterize its activity. To examine if the colicin could act upon closely related *E. coli*, we induced colicin production in wild-type MP1 by UV light and then spot plated serial dilutions of the cell lysate on soft agar plates containing the laboratory K-12 strain MG1655. The zone of inhibition was determined by scoring for either clear zones for complete inhibition or turbid zones for partial growth inhibition. In the absence of UV light exposure, turbid growth was only observed with the undiluted lysate, suggesting detectable but low basal levels of colicin production (Figure 1C). For cell lysate from UV-exposed MP1, however, clear zones of inhibition were seen up to a dilution of 10^3^, suggesting significant production of active colicin upon induction of DNA damage. We also explored the spectrum of activity of the MP1 colicin against a broader range of isolates (Table S1). We observed activity against the *E.coli* Nissle 1917 strain, but not against other *E. coli* strains, including group A and B2 commensals, or more distantly related pathogens including *Salmonella*, *Vibrio cholerae,* and *Klebsiella*. Collectively, these data demonstrate that MP1 carries an inducible, competent, and narrow-spectrum colicin, which could plausibly have a role in MP1 colonization and survival in its natural environmental niche, the mammalian gut. 

### 3.2. Sustained Colonization in Healthy Murine Gut Does Not Require Colicin Production 

Previous studies have reported the importance of colicins for bacterial survival within the inflamed mammalian gut [31,47]. These studies, however, do not address whether colicins are critical effector proteins used by commensals to initiate and maintain colonization in the absence of intestinal perturbations. The MP1 model of colonization is unique in that colonization can be achieved in the absence of continuous antibiotic treatment. Here, the MP1 model has a brief streptomycin pre-treatment to minimize colonization resistance followed by a 24 h washout period where streptomycin is removed from the water and not replaced prior to strain inoculation. The mice are then inoculated by oral gavage with the bacterial strain of interest and feces is collected at various time points to assess the efficiency and stability of strain colonization with time. Notably, in control experiments, several days following the brief streptomycin treatment the normal flora rebounds, making this system a reliable model for the unperturbed or minimally perturbed mammalian gut [48]. The validated MP1 model has been used to demonstrate a key role for bacterial nitrogen production in altering the gut microbiome, a role of the SOS response during *E. coli* colonization, and the importance of two-component signaling systems in sustained colonization [22,32,49].

**Figure 2:**
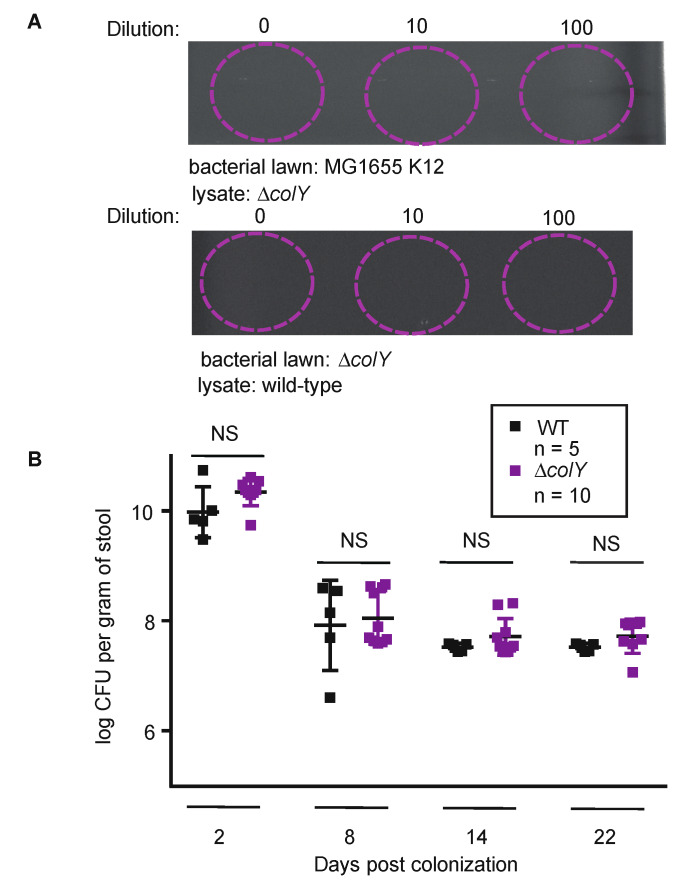
Colicin-deficient strain can establish colonization in healthy mouse gastrointestinal tract. (**A**) Shown is the inhibition halo of ∆*colY* strain exposed to 50 J/m^2^ and extracted lysate spot plated on a lawn of *E. coli* MG1655-K12 and inhibition halo of wild-type strain exposed to 50 J/m^2^ and extracted lysate spot plated on a lawn of ∆*colY* strain. Dilutions of cell lysates were made and indicated. Circles indicate location of spotted lysate. (**B**) Groups of 6- to 8-week-old C57BL/6 male mice were orally inoculated with either the wild-type strain (black squares) or the ∆*colY* strain (purple squares). On the days indicated, fecal samples were collected and CFU counts were determined. Each square represents one mouse and the limit of detection was 10^3^. Significant *p* values are noted (NS, not significant) and were calculated using a two-tailed unpaired Student’s *t* test. If colonies were too numerous to count on a plate, the corresponding animal was excluded from the data on that day.

To investigate the role of colicins in our experimental system, we used a previously constructed pair of wild-type MP1 derivatives containing either mCherry or GFP under the control of a *tet* promoter [32]. We further engineered the GFP-expressing strain by replacing the colicin gene, *colY*, with a kanamycin-resistance cassette [33]. In this variant, only the colicin gene was disrupted, leaving the lysis and immunity genes intact. We validated that our colicin-deficient (∆*colY*) strain did not produce an UV-inducible colicin and that the strain remained resistant to the MP1 colicin, indicating that immunity was intact (Figure 2A). Therefore, using this strain we could specifically interrogate whether only the lack of colicin production would impact the ability of MP1 to sustain colonization. 

Taking advantage of the well-established MP1 mouse model, we orally inoculated C57BL/6 male mice with either the wild-type *mcherry*-marked strain or the ∆*colY gfp*-marked strain. With the aim of understanding colonization dynamics in these solo-colonization experiments, we collected feces at the beginning of the experiment until termination of the experiment at 22 days. At each time point, feces were normalized by weight, serially diluted, and plated onto LB agar containing tetracycline, which permits selection of the MP1 strains and induces the expression of either *gfp* or *mcherry*. Across time points, the colonization levels of the ∆*colY* strain and the wild-type strain showed no statistical difference (Figure 2B). For both groups of mice, there was an initial drop in colony forming units (CFU) after week one, but for both the wild-type strain and the ∆*colY* strain the CFUs stabilized at ~10^7^ per gram of stool for the duration of the experiment. These data suggest that MP1 colonizing its natural niche environment may not require colicin production for initiating and maintaining colonization. 

**Figure 3:**
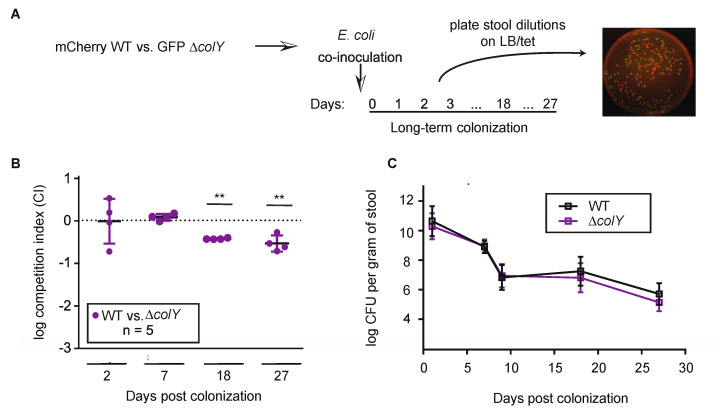
Competition of ∆*colY* in the adult mouse gastrointestinal tract. (**A**) Schematic of the competition protocol. Mice were inoculated in a 1:1 ratio of either strain and fecal samples were collected at various days post-inoculation. Fecal samples were serially diluted and plated onto LB/tetracycline plates. A representative plate is shown. (**B**,**C**) Groups of 6- to 8-week-old C57BL/6 mice were co-inoculated. (**B**) Log competitive index (CI) was calculated by taking the ratio of the output colonies normalized to the input ratio. Each circle represents one specific animal. (**C**) CFU counts per gram of feces for the competition experiment. Each square represents the mean and standard deviation from all individual mice. Limit of detection was 10^2^. Significant *p* values are noted (** *p* > 0.005) and were calculated using a one sample t-test. If colonies were too numerous to count on a plate, the corresponding animal was excluded from the data for that day.

Given that MP1 is a host-adapted strain, we posited that it may have evolved to efficiently contend with ongoing environment perturbations as a consequence of living in a dynamic gastrointestinal tract. We therefore considered that more subtle fitness advantages might be masked in solo-colonization experiments and could potentially manifest when our colicin-proficient and colicin-deficient strains were in direct competition for an intestinal niche. Toward this end, we orally inoculated C57BL/6 male mice with equal mixtures of ∆*colY gfp-*marked strain and the wild-type *mcherry*-marked strain. We quantified the initial inoculum and collected feces continuously throughout the experiment (Figure 3A). At each time point, we calculated the competitive index (CI) in order to quantify the relative fitness of the ∆*colY* strain relative to the wild-type strain. The CI was calculated by taking the ratio of input CFU counts to output CFU counts. In this competition experiment between the wild-type strain and the ∆*colY* strain, up to 7 days post-inoculation there was no relative colonization defect between the strains (Figure 3B, log CI = 0.061). By day 27, a mild colonization defect was observed whereby the wild-type strain outcompeted the colicin deficient strain by ~3-fold (log CI = −0.51, *p* = 0.003). Over the course of this experiment the overall CFU suggest stable maintenance of both strains, although the levels are reduced relative to the solo-colonization experiment (Figure 3C). Our data indicate that both strains can stably co-colonize the murine gut, and suggest that in this mixed population of commensals, the lack of colicin production has only a small effect on long-term maintenance of colonization.

### 3.3. Sustained Colonization in Healthy Murine Gut Does Not Require Colicin Immunity

**Figure 4:**
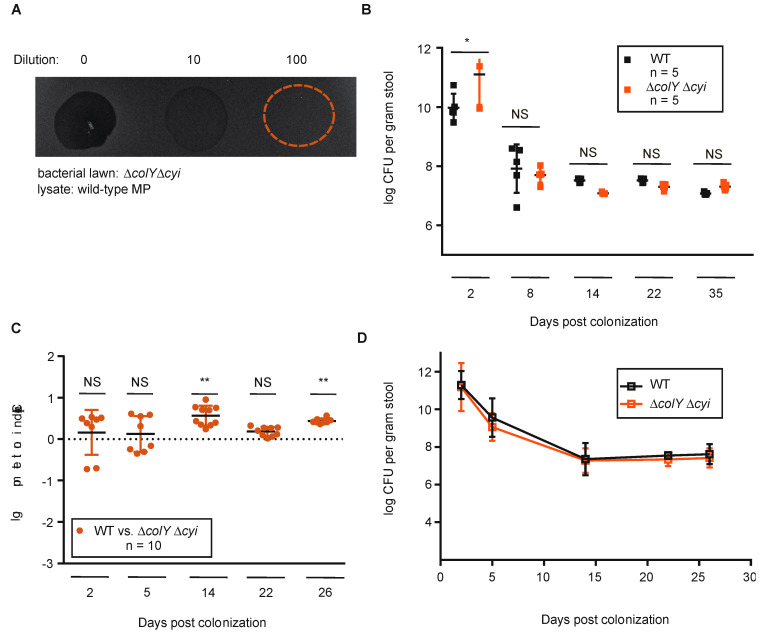
Characterization and colonization of the adult mouse gut by a colicin-sensitive strain. (**A**) Inhibition halo of wild-type strain exposed to 50 J/m^2^ and extracted lysate spot plated on a lawn of ∆*colY* ∆*cyi* strain. Dilutions of cell lysates were made and indicated above. Circles indicate location of spotted lysate. (**B**) Groups of 6- to 8-week-old C57BL/6 mice were inoculated with the colicin wild-type strain or the ∆*colY* ∆*cyi* strain. Significant *p* values are noted (NS, not significant. * *p* > 0.05) and were calculated using a two-tailed unpaired Student’s *t* test. (**C**,**D**) Groups of 6- to 8-week-old C57BL/6 mice were co-inoculated. (**C**) Log competitive index (CI) was calculated by taking the ratio of the output colonies normalized to the input ratio. Each circle represents one specific animal. Significant *p* values are noted (NS, not significant, ** *p* > 0.005) and were calculated using a one sample *t*-test. (**D**) CFU counts per gram of feces for the competition experiment. Each square represents the mean and standard deviation from all individual mice. Limit of detection was 10^3^. If colonies were too numerous to count on a plate, the corresponding animal was excluded from the data for that day.

Evaluation of the ∆*colY* strain afforded us the opportunity to specifically test the effect of colicin production during sustained colonization. However, given that the ∆*colY* strain is immune to the MP1 colicin, this experimental setup did not permit us to ascertain whether the colicin production and immunity pair together offers advantages to MP1 during competition. Since the ∆*colY* strain is resistant to its colicin, it is conceivable that the engineered ∆*colY* strain was benefiting from the colicin production of the wild-type strain, thus masking any fitness defect that might exist. Although there was no detectable difference between the wild-type and ∆*colY* strain during solo-colonization experiments, it is possible that there are different and significant fitness requirements during direct competition in the mammalian gut. To address these possibilities, we further manipulated the ∆*colY* strain to also delete the immunity gene, *cyi*. We confirmed that the ∆*colY* ∆*cyi* does not produce an UV-inducible colicin and that this engineered strain is now sensitive to MP1 colicin (Figure 4A). 

Before proceeding to competition experiments, we first evaluated whether colicin sensitivity had an impact on solo-colonization by MP1. Either the wild-type or the ∆*colY* ∆*cyi* strain were orally inoculated into C57BL/6 male mice and evaluated for total CFU over a 4-week period. In this setting, the ∆*colY* ∆*cyi* strain colonized to a similar extent and with comparable kinetics relative to the wild-type strain; the CFU counts stabilized in the gut within 1 week and remained stable for >4 weeks at ~10^7^ per gram of stool (Figure 4B). The absence of any statistical difference between the colonization suggests that the ability of MP1 to initiate and sustain colonization is independent of both colicin production and resistance. 

To next evaluate whether competition would elicit differences between the two strains, we orally inoculated C57BL/6 male mice with equal mixtures of the ∆*colY* ∆*cyi* strain and the wild-type strain, tracked CFU in feces over time, and calculated the competitive index. As with solo-colonization, there was an initial drop in CFUs with the levels plateauing around day 14 and remaining stable. At each timepoint evaluated, the wild-type strain and the ∆*colY* ∆*coli* strain were found in similar amounts, which was reflected in the log CI values (Figure 4C,D). Specifically, by day 14 a small colonization advantage was observed in the ∆*colY* ∆*cyi* strain relative to the wild-type strain (log CI = 0.54, *p* = 0.002). However, the mild colonization advantage for the ∆*colY* ∆*cyi* strain was not consistently significant over time. By the termination of the experiment there was only roughly a 3-fold advantage to the ∆*colY* ∆*cyi* strain (log CI = 0.42, *p* = 0.003). Taken together, our results suggest that colicin production and resistance are not required for a commensal to establish and sustain colonization and does not augment niche competition in a healthy intestinal environment. Our results suggest that there may be a slight advantage to not harboring both a functional colicin and the immunity gene during commensal gut colonization. 

### 3.4. Withstanding Strain Invasion in the Murine Gut Does Not Require Colicin Production

**Figure 5:**
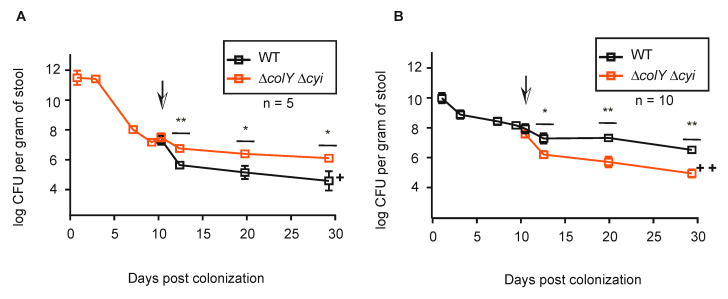
Commensal strain invasion. (**A**,**B**) Groups of 6- to 8-week-old C57BL/6 mice were inoculated with either the wild-type strain or the ∆*colY* ∆*cyi* strain. On day 9, as represented by the down arrow, the mice were inoculated with the wild-type strain (**A**) or the ∆*colY* ∆*cyi* strain (**B**). Each square represents the mean and standard deviation from the mean of all individual mice. Limit of detection was 10^2^. If colonies were too numerous to count on a plate, the corresponding animal was excluded from the data for that day. + indicates the wild-type strain was not recoverable from one mouse; ++ indicates the ∆*colY* ∆*cyi* strain was not detected from two mice by the termination of the experiment. Significant *p* values are noted (* *p* > 0.05, ** *p* > 0.005) and were calculated using a two-tailed unpaired Student’s *t* test.

Our work thus far has demonstrated that MP1′s colicin is not critical for stable gut colonization and does not provide a significant fitness benefit during direct niche competition in a healthy gut environment. However, resilience of commensals in the microbiome also depends upon their ability to withstand invasion of their niche by a competing strain. Therefore, we wanted to explore whether colicins aid in MP1′s ability to withstand niche invasion by another commensal. To mirror that scenario, mice were pre-colonized with the *gfp-*marked ∆*colY* ∆*cyi* strain and at day 9 post-inoculation were fed *mcherry*-marked wild-type strain (~10^6^ CFU/mouse). In contrast to the co-inoculating experiments, the wild-type strain failed to sustain colonization in mice pre-colonized with ∆*colY* ∆*cyi*. By day 3 post-inoculation with the wild-type strain, there was an initial drop in CFU for both strains, but the drop in CFU for the wild-type strain was ~10-fold greater than the drop in CFU for the ∆*colY* ∆*cyi* strain (Figure 5A, *p* > 0.007). By day 10 post-wild-type inoculation, the wild-type strain dropped to ~10^4^ CFUs whereas the ∆*colY* ∆*cyi* strain remained stable at ~10^6^ CFUs (*p* > 0.04). At the termination of the experiment, the wild-type strain was only recovered in two out of the five starting mice and the ∆*colY* ∆*cyi* strain remained stable at ~10^6^ CFUs (*p* > 0.05). Therefore, the ∆*colY* ∆*cyi* strain was able to withstand invasion and prevent colonization by the wild-type strain, suggesting that colicin production and resistance is not critical for a commensal to outcompete invading strains. 

Since pre-colonization of the ∆*colY* ∆*cyi* strain prevented the wild-type stain from robustly colonizing the gastrointestinal tract, we next wanted to assess whether the wild-type strain could prevent the ∆*colY* ∆*cyi* strain from establishing and maintaining colonization. To do this, mice were pre-colonized with the *mcherry-*marked wild-type strain and at day 9 post-inoculation were fed *gfp*-marked ∆*colY* ∆*coli* (~10^7^ CFU/mouse). Again, by day 3 post-inoculation with the ∆*colY* ∆*cyi* strain, there was an initial drop in CFUs for both strains, but in this experiment the drop in CFUs for the invading ∆*colY* ∆*cyi* strain was ~10-fold (10^6^) whereas the wild-type strain dropped by ~5-fold (~10^7^) (Figure 5B). By day 10 post-∆*colY* ∆*cyi* strain inoculation, the pre-colonized wild-type strain stabilized at 10^7^ CFU whereas the ∆*colY* ∆*cyi* strain continued to drop in CFUs eventually stabilizing at ~10^5^ (*p* > 0.002). As with the reverse scenario, at the termination of the experiment the wild-type strain remained stable at ~10^6^ CFU and the ∆*colY* ∆*cyi* strain was unable to robustly maintain colonization (*p* > 0.001). Under these conditions, the ∆*colY* ∆*cyi* strain did not displace the wild-type strain and was unable to sustain colonization levels. Taken together, our data suggest that the ability to withstand strain invasion may be independent of colicin production and resistance. 

## 4. Discussion

The abundance and diversity of colicin-producing bacteria suggest that colicins could be important factors for successful bacteria survival in complex dynamic microbial communities. Attempts to elucidate the specific role of colicins has mostly been limited to mathematical models, well-defined laboratory settings, or perturbed environmental systems. These studies have been invaluable for deciphering the molecular mechanism of colicins and for providing models that suggest colicin production can impact interactions with the competing microbes and the environment. However, these previous studies have offered more limited insight into the importance of colicin production in the context of a natural, unperturbed environment. The mammalian gut, composed of a high density of competing bacteria interacting with the host, makes for an ideal setting to elucidate the importance of colicin production in a complex microbial environment. In this study, we used a well-established C57BL/6 mouse model to address whether colicins are important for colonization, niche competition, and strain invasion using a natural colicin-producing commensal strain. Significantly, our results demonstrate that colicin production is not essential for sustained colonization of a commensal *E. coli* strain in a healthy mouse gut with an intact gut microbiome. During solo-colonization experiments, both a colicin-deficient strain and a colicin-sensitive strain sustained colonization levels statistically similar to the wild-type colicin-producing strain. Interestingly, the wild-type strain showed no competition advantage over either a colicin-deficient strain or a colicin-sensitive strain. Further, the ability of a pre-colonized strain to withstand niche invasion by another strain was independent of colicin production and sensitivity. This is the first report demonstrating that colicins are not required for sustained colonization of a commensal *E*. *coli* strain inhabiting its natural niche in the healthy murine gut. 

Successful bacteria colonization involves both initiating and sustaining colonization [8]. In order to fully capture both aspects of colonization we took a kinetic approach and monitored bacterial burden across time throughout each experiment. With this approach, we were able to conclude that colicin production is not essential for a commensal to establish itself in its environmental niche nor to maintain itself in that niche. It is possible, however, that if the duration of the colonization experiments had been extended there would have been a reduction in colicin-deficient colony counts. In one prior experimental setup, the benefit of colicin production was revealed only after ~3 months of colonization [28]. However, that experiment was done in the context of an altered microbiome with the mice being continually fed streptomycin for the duration of the experiment, which could be associated with mild inflammation [47]. It is possible that in the setting of mild inflammation colicin production has a more critical role for commensal survival. Further, the colony counts in our experiments stabilized over time, suggesting the duration of our experiments appropriately captured stable colonization.

For a commensal microbe, the gut is a complex environment with ongoing interactions with surrounding microbes. To examine the importance of colicin production during direct competition over the same niche, we performed two separate competition experiments. In one competition experiment we competed the wild-type strain against a colicin-deficient strain to specifically interrogate the role of colicin production. In the second competition experiment we competed the wild-type strain against a colicin-sensitive strain to examine directly whether colicin sensitivity would result in a fitness disadvantage. In both experiments each strain was recovered in nearly equal numbers throughout the experiment. Our data suggest that for a commensal in its adapted environment, potentially costly defense mechanisms such as colicins are not essential. This conclusion is further supported by the pre-colonization experiments in which either a pre-colonized wild-type strain or pre-colonized strain that is both colicin deficient and sensitive was able to prevent robust colonization of an invading strain. It is possible that maintenance of microbial diversity and fitness in homeostatic environments may rely on alternative mechanisms besides colicin production. Several studies have demonstrated that established commensals can outcompete other microbes via other mechanisms including physical and nutrient exclusion [7,8,50]. Given the complex nature of the gut microbiome, it is probable that multiple mechanisms are involved for a commensal to maintain colonization in its intestinal niche. Further, it is possible that in homeostatic environments, the expression of colicins may in fact come with costs. Colicin machinery can be energetically costly to bacteria and, specifically with pore-forming colicins such as MP1′s colicin, the gene encoding the immunity protein is typically constitutively expressed [12]. Our results could align with this conclusion because when the wild-type strain was competed against the colicin-sensitive strain, the colicin-sensitive strain showed a mild colonization advantage at select time points.

The results presented here suggests colicin dynamics are closely linked to the surrounding environment. Collectively, our data demonstrate that in a healthy, minimally perturbed gut environment colicins do not contribute to commensal survivability whereas previous reports have clearly demonstrated an important role of colicins for bacterial survival in the setting of gut inflammation [30,51,52]. Specifically, previous work using a streptomycin-treated mouse model revealed that when a mouse is pre-colonized with a colicin sensitive strain, introduction of a colicin-producing strain can displace the sensitive strain [29]. In our parallel experiment, mice were pre-colonized with a colicin-deficient and -sensitive strain and when a colicin-producing strain was introduced, it was unable to invade and displace the existing strain (Figure 5A). Taken together, these results suggest that colicin dynamics depend upon the surrounding gut environment or on the given strain being investigated. 

We also established that MP1 colicin production is SOS-dependent and increased in the presence of genotoxic stress. Genes regulated by the SOS response are induced in a highly organized chronological manner where the first genes to be induced are those involved in error-free repair and the last genes to be induced are those involved in error-prone repair [42,53]. It is hypothesized that induction of error-prone repair is reserved for when there is extensive or sustained DNA damage. Our analysis of the MP1 colicin promoter indicates that it would be likely induced later in the SOS response, suggesting that colicin production is reserved for times of sustained or considerable DNA damage [41]. These results are particularly important to consider in the context of our prior work demonstrating that the SOS response is important for sustained colonization of MP1 in a natural gut environment [22]. In that report, an SOS-off mutant had a colonization defect relative to a wild-type strain. While we speculated that the loss of colicin production might contribute to the reduced fitness of the SOS-off mutant, the results presented here suggest that alternative SOS-associated effectors other than the MP1 colicin are more likely to be responsible for the compromised in vivo fitness of the SOS-off MP1 strain. There are several potential explanations to account for an observed fitness defect with an SOS-off MP1 strain and the absence of a fitness defect with the colicin-deficient strain. From a regulatory perspective, it is possible that the genotoxic stress experienced during colonization is not enough to activate late SOS-induced genes, but enough to activate early SOS-induced genes which account for the phenotype. Additionally, given the IcsR binding site in the promoter, it is possible that despite SOS activation, colicin expression remains repressed by IcsR in the unperturbed host environment. Previous studies have speculated that dual repression of colicins by both LexA and IscR permits colicin expression to be reserved as a last resort mechanism [41]. Lastly, it is conceivable that MP1 colicins are produced, but that given their narrow spectrum they have no major impact on the neighboring microbes that contribute to the success or failure of stable colonization.

Our model using MP1 colonization of the murine gut has distinctive advantages in that MP1 is a natural colonizer and establishes colonization levels that are close to those observed for *E. coli* in the unperturbed murine gut. At the same time, there are two aspects of our study which might limit the generalizability of our conclusions. First, our analysis of MP1 colicin indicated that it is active against non-immune MP1 partners, MG1655, and *E. coli* Nissle, but is not more broadly active. Although our study validates that natural colicin producers are not universally dependent upon their colicins, it is possible that other natural colonizers producing broader-acting colicins may be more dependent upon their production. Second, we utilized a brief, but non-sustained, streptomycin treatment prior to colonization with MP1, in order to open up a niche that can be reliably colonized to reproducible levels. While prior work has established that the normal flora rebounds within five days of treatment [48,54] and that our model best represents the unperturbed gut, it is feasible that our minimal perturbations prior to colonization could alter the microbiome in a manner that impacts our observations.

Overall, the results of our study combined with previous data exploring the role of colicins in alternative systems suggest a model where the fitness afforded by colicin production may depend upon the environmental condition. MP1 is a natural *E. coli* isolate of the murine gut and, by extension, when it is colonizing a healthy mouse gut the environmental stressors might be minimally perturbing. In light of our previous study demonstrating a role for the SOS response during MP1 colonization of the murine gut, it is possible that basal genotoxic stress is present, requiring some activation of the SOS response, but the genotoxic stress is not extensive enough to induce or require some of the more potent effectors, such as colicins.

Importantly, the results of this study may help contribute towards defining the role of colicin-producing bacteria as probiotics that may prevent infection by enteric pathogens. Our data suggest that colicin production might be activated only in the setting of dysbiosis, and thus the therapeutic benefit of colicins might be enhanced if utilized during the initial period of dysbiosis. We speculate that in the absence of exogenous perturbations, competition between bacterial communities is not as fierce as previously imagined, but instead these communities exist in a delicate equilibrium that does not require routine activation of the full arsenal of bacterial defense mechanisms. Our study highlights a role for exploring effectors in various environments, including those most closely mimicking natural systems, when considering the dynamics of the microbiome.

## Supplementary Materials

The following are available online at https://www.mdpi.com/2076-2607/8/5/664/s1, Figure S1: Schematic of MP1 plasmid map, Figure S2: SOS regulation of colicin promotor, Table S1: Characteristic and sensitivity profile of strains used to test activity of MP1 colicin. Supplementary References.
