# Supplementary material for: Deciphering the Role of Colicins during Colonization of the Mammalian Gut by Commensal E. coli
Source: Microorganisms. 2020 May 2;8(5):664. doi: 10.3390/microorganisms8050664 (PMC7284606; doi:10.3390/microorganisms8050664)
Supplement: Supplementary file 1 [file microorganisms-08-00664-s001.pdf]

*microorganisms*

*Supporting Information*

**Deciphering the role of colicins during colonization of the mammalian gut by commensal *E. coli***

**Amanda N. Samuels [1,2], Manuela Roggiani [3], Kathryn A. Smith [3,4], Jun Zhu [2], Mark Goulian [3], and Rahul M. Kohli [5,*]**

[1] Department of Veterinary Medicine, University of Pennsylvania, Philadelphia, Pennsylvania, USA; samuelsa@upenn.edu
[2] Graduate Group on Cell and Molecular Biology, Perelman School of Medicine, University of Pennsylvania, Philadelphia, Pennsylvania
[3] Department of Biology, School of Arts and Science, University of Pennsylvania, Philadelphia, Pennsylvania
[4] Department of Biology, Solenis LLC., Wilmington, Delaware
[5] Department of Medicine, Perelman School of Medicine, University of Pennsylvania, Philadelphia, Pennsylvania

* Correspondence: rkohli@pennmedicine.upenn.edu; Tel.:+ 01-(215)-573-7523

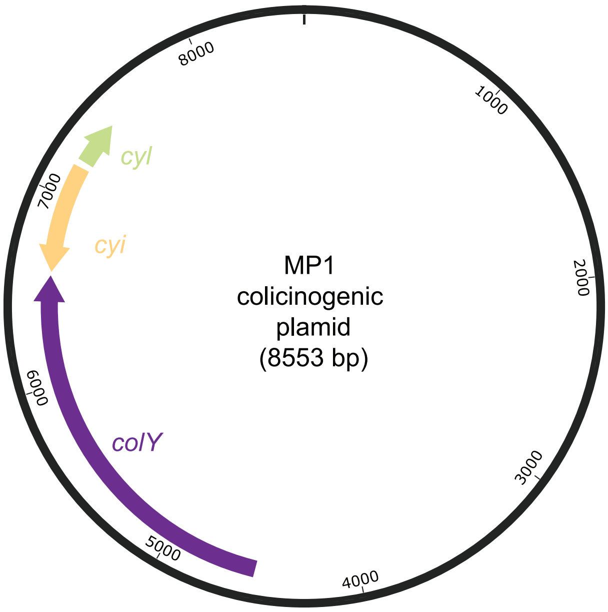


**Figure S1.** Schematic of MP1 plasmid map with annotated colicin (*colY*), immunity (*cyi*), and lysis (*cyl*) genes.

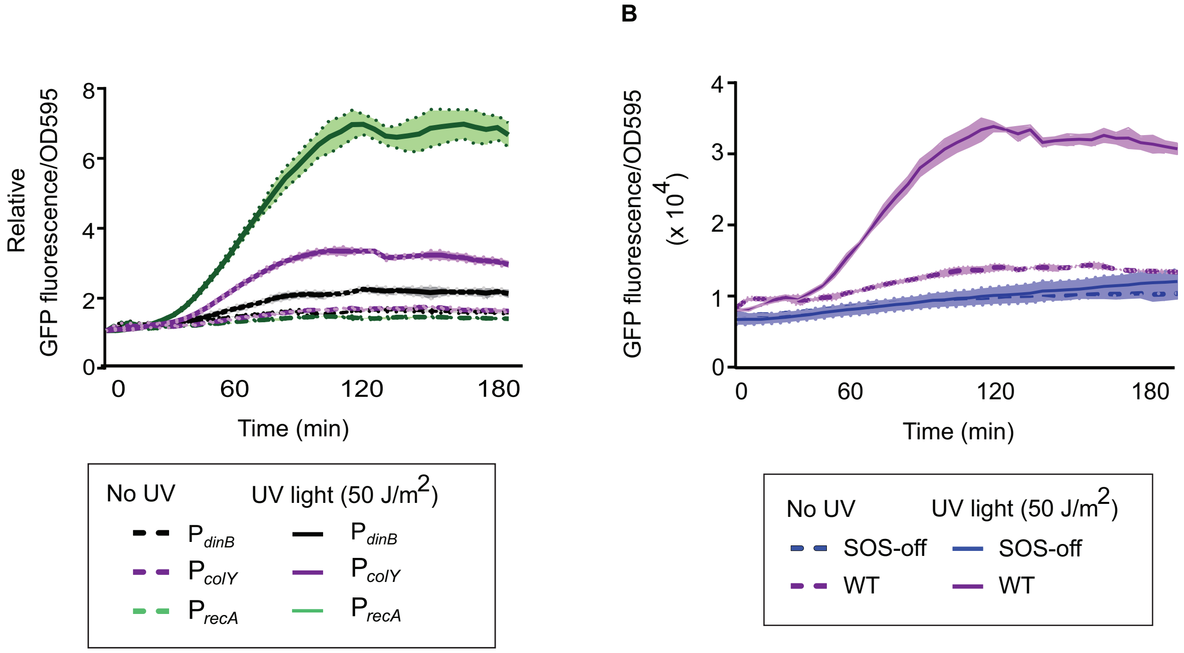


**Figure S2.** SOS regulation of colicin promoter. (A) The promoter activity of *recA* is compared to the promoter activity of *colY* and *dinB*. GFP induction for $P_{recA}$ or $P_{colY}$ is indicated by the green and purple colors, respectively. (B) The promoter activity *colY* is dependent upon LexA cleavage. SOS-off represents a strain that has an inactivated LexA protein that prevents activation of the SOS response. For both experiments the time-dependent induction of GFP is represented as fluorescence intensity normalized to optical density at 595 nm at the start of the experiment. The error bands show the standard deviations of results from four independent biological replicates for each condition.

| Strain | Relevant characteristics | Sensitivity profile | Citation |
|---|---|---|---|
| ***Vibrio Cholerae* C6076** | El Tor, O1 | Resistant | [55] |
| ***Klebsiella pneumonia* A2312NM** | ST-11 serogroup | Resistant | [56] |
| ***S. typhimuirum* 14028s** | Outgroup GC1 | Resistant | [57] |
| ***S. typhimurium* AJB3** | | Resistant | This study |
| ***S. Gallinarum* ATCC700623** | Biovar Gallinarum | Resistant | This study |
| ***S. Newport* SL254** | S. Newport IIC | Resistant | This study |
| ***E. coli E2348/69*** | Group B2, EPEC | Resistant | [58] |
| ***E. coli CFT073*** | Group B2, UPEC | Resistant | [59] |
| ***E. coli Nissle 1917*** | Group B2, Commensal | Sensitive | [60] |
| ***E. coli 042*** | Group D, EAEC | Resistant | [61] |
| ***E. coli* HS** | Group A, Commensal | Resistant | [62] |
| ***E. coli* O157:H7 EC4115** | Group B, EHEC | Resistant | [63] |
| **K12 MG1655** | Group A, Commensal | Sensitive | *E. coli* Genetic Stock Center, CGSC no. 7740 |
| **TW10509** | *Clade* I, ETEC | Resistant | [64] |

**Table S1.** Characteristics and sensitivity profile of strains used to test the activity spectrum of MP1 colicin

## Supplementary References

55. Joelsson, A.; Liu, Z.; Zhu, J. Genetic and phenotypic diversity of quorum-sensing systems in clinical and environmental isolates of *Vibrio cholerae*. *Infect Immun* **2006,** *74,* 1141–1147.

56. Hao, G.; Chen, A.I.; Liu, M.; Zhou, H.; Egan, M.; Yang, X.; Kan, B.; Wang, H.; Goulian, M.; Zhu, J. Colistin resistance-mediated bacterial surface modification sensitizes phage infection. *Antimicrob Agents Chemother* **2019,** *63,* e01605-19.

57. Jarvik, T.; Smillie, C.; Groisman, E.A.; Ochman, H. Short-Term signatures of evolutionary change in the *Salmonella enterica* Serovar Typhimurium 14028 genome. *J Bacteriol* **2010,** *192,* 560–567.

58. Iguchi, A.; Thomson, N.R.; Ogura, Y.; Saunders, D.; Ooka, T.; Henderson, I.R.; Harris, D.; Asadulghani, M.; Kurokawa, K.; Dean, P.; Kenny, B.; Quail, M.A.; Thurston, S.; Dougan, G.; Hayashi, T.; Parkhill, J.; Frankel, G. 2009. Complete genome sequence and comparative genome analysis of Enteropathogenic *Escherichia coli* O127 : H6 Strain E2348 / 69 . *J Bacteriol* **2009,** *191,* 347–354.

59. Mobley, H.L.; Green, D.M.; Trifillis, A.L.; Johnson, D.E.; Chippendale, G.R.; Lockatell, C.V.; Jones, B.D.; John, W. Pyelonephritogenic *Escherichia coli* and killing of cultured human renal proximal tubular epithelial cells : role of hemolysin in some strains. Infect Immun **1990,** *58,* 1281–1289.

60. Grozdanov, L.; Raasch, C.; Schulze, J.; Sonnenborn, U.; Gottschalk, G.; Hacker, J.; Dobrindt, U. Analysis of the genome structure of the nonpathogenic probiotic *Escherichia coli* strain Nissle 1917. *J Bacteriol* **2004,** *186,* 5432–5441.

61. Nataro, J.P.; Baldini, M.M.; Kaper, J.B.; Black, R.E.; Bravo, N.; Levine, M.M. Detection of an adherence factor of enteropathogenic *Escherichia coli* with a DNA probe. *J Infect Dis* **1985,** *1,* 152560–565.

62. Rasko, D.A.; Rosovitz, M.J.; Myers, G.S.A.; Mongodin, E.F.; Fricke, W.F.; Gajer, P.; Crabtree, J.; Sebaihia, M; Thomson, N.R.; Chaudhuri, R.; Henderson, I.R.; Sperandio, V.; Ravel, J. The pangenome structure of *Escherichia coli* : comparative genomic analysis of E . coli commensal and pathogenic isolates. *J Bacteriol* **2008,** *190,* 6881–6893.

63. Eppinger, M.; Mammel, M.K.; Leclerc, J.E.; Ravel, J.; Cebula, T.A. Genomic anatomy of *Escherichia coli* O157 : H7 outbreaks. *PNAS* **2011,** *108,* 20142–20147.
